# Inflammatory and Immune Responses during SARS-CoV-2 Infection in Vaccinated and Non-Vaccinated Pregnant Women and Their Newborns

**DOI:** 10.3390/pathogens12050664

**Published:** 2023-04-29

**Authors:** Paola Zelini, Piera d’Angelo, Federica Zavaglio, Ehsan Soleymaninejadian, Liliana Mariani, Francesca Perotti, Mattia Dominoni, Stelvio Tonello, Pierpaolo Sainaghi, Rosalba Minisini, Daria Apostolo, Daniele Lilleri, Arsenio Spinillo, Fausto Baldanti

**Affiliations:** 1 Molecular Virology Unit, Microbiology and Virology Department, Fondazione IRCCS Policlinico San Matteo, 27100 Pavia, Italy; 2 Obstetrics and Gynecology Unit, Fondazione IRCCS Policlinico San Matteo, 27100 Pavia, Italy; 3 Department of Clinical, Surgical, Diagnostics and Pediatric Sciences, University of Pavia, 27100 Pavia, Italy; 4 Immunoreumatology Laboratory, Center for Translational Research on Autoimmune and Allergic Disease-CAAD, University of Piemonte Orientale, 28100 Novara, Italy; 5 Internal Medicine Laboratory, Department of Translational Medicine, University of Piemonte Orientale, 28100 Novara, Italy

**Keywords:** pregnancy, SARS-CoV-2, immunity, vaccine

## Abstract

Background. Pregnant women are more susceptible to severe disease associated with SARS-CoV-2 infection. We performed a prospective study to analyze the inflammatory and immune profile after SARS-CoV-2 infection occurring in vaccinated or non-vaccinated pregnant women and their newborns. Methods. Twenty-five pregnant women with SARS-CoV-2 infection were enrolled, and sixteen cord blood samples were obtained at delivery. Results. We observed that IL-1β, TNF-α, Eotaxin, MIB-1β, VEGF, IL-15, IL-2, IL-5, IL-9, IL-10 and IL-1ra levels were significantly higher in vaccinated than non-vaccinated mothers. Furthermore, the newborns of the vaccinated mothers produced higher levels of IL-7, IL-5 and IL-12 compared to the newborns of non-vaccinated mothers. Anti-Spike (S) IgG levels were significantly higher in all vaccinated mothers and their newborns compared to the non-vaccinated group. We found that 87.5% of vaccinated women and 66.6% of non-vaccinated women mounted an S-specific T-cell response quantified by ELISpot assay. Moreover, 75.0% of vaccinated mothers and 38.4% of non-vaccinated mothers showed S-specific CD4^+^ T-cell proliferative response. The T-helper subset response was restricted to CD4^+^ T_h_1 in both vaccinated and non-vaccinated women. Conclusion. A higher level of cytokines, IgG antibodies and memory T cells was noted in the vaccinated women. Furthermore, the maternal IgG antibody trans-placental transfer occurred more frequently in vaccinated mothers and may protect the newborn.

## 1. Introduction

Pregnancy is a state of altered immunity, causing pregnant women to be more vulnerable to viral infections, including SARS-CoV-2 infection. Data from previous pandemics, such as those caused by SARS, influenza virus H1N1 [1] and MERS [2], show that pregnant women are more susceptible to serious illness with adverse outcomes and display greater mortality rates than the general population. However, a recent meta-analysis demonstrates that pregnant women with COVID-19 have similar clinical characteristics and outcomes as the non-pregnant population [3]. Despite the limited evidence of vertical transmission [3], infected women are at significantly higher risk for cesarean delivery, preterm birth and adverse neonatal outcomes [4,5,6,7] compared to the general pregnant population. Moreover, increased levels of pro-inflammatory cytokines, IL-8, IL-10 and IL-15, have been observed in the circulation of pregnant women with SARS-CoV-2 infection and their neonates, even in the absence of placental infection [8]. Gee and colleagues showed an increase in cytokines in cord plasma following the presence of an inflammatory profile in the mothers but also altered immune cell functionality in neonates exposed to SARS-CoV-2 at any point during gestation [9]. Notwithstanding their higher risk, pregnant and lactating women were not included in any initial COVID-19 vaccine trials, although the first vaccine trial with pregnant women began in February 2021 (Pfizer/BioNTech, ClinicalTrials.gov identifier: NCT04754594). The preliminary findings did not show additional complications among pregnant women who received BNT162b2 (Pfizer/BioNTech) and mRNA-1273 (Moderna) compared to non-pregnant persons [10,11,12,13]. Moreover, the proportions of adverse pregnancy and neonatal outcomes (i.e., preterm birth, congenital anomalies and neonatal death) among mothers with completed pregnancies appeared to be similar to the published incidences in pregnant populations studied before the COVID-19 pandemic [14,15,16].

Numerous studies have reported the effects on the fetus following vaccination in pregnancy [17,18]. The maternal antibodies increased by the vaccination during pregnancy cross the placenta and are transferred in the umbilical cord blood at birth [19,20], remaining detectable in the blood of more than half of newborns at 6 months [21]. In addition, many studies indicate that anti-SARS-CoV-2 IgG and IgA antibodies were transmitted to newborns through vaccinated mother’s milk, resulting in a probable protective role [22,23]. In this prospective study, we evaluated the inflammatory and immune profile in pregnant women vaccinated and non-vaccinated during SARS-CoV-2 infection.

## 2. Methods

### 2.1. Study Design

A prospective observational study was conducted to evaluate the cytokine, antibody and T-cell responses in vaccinated and non-vaccinated SARS-CoV-2-infected pregnant women and their newborns. The study included 25 pregnant women (median age 32 years; range 19–43) enrolled between January 2022 and February 2022 at the Obstetrics and Gynecology Clinics of Fondazione IRCCS Policlinico San Matteo (Table 1). All pregnant women were hospitalized to treat preterm labor, hypertension preeclampsia and cholestasis or because they were close to delivery (median gestational weeks: 39.0; range 16.0–42.0).

A total of 25 pregnant women were recruited during the study period. Age > 18, two doses of the vaccine or non-vaccine administration were the inclusion criteria. At the time of enrollment, all of them were diagnosed with SARS-CoV-2 infection via RT-PCR by taking a nasal swab from each of them. Blood and serum samples were obtained from all women subsequently. In addition, all information on the SARS-CoV-2 vaccine administered to the patients was collected.

Thus, 9/25 (36%) pregnant women received two doses of vaccine before conception (median days before diagnosis of infection: 100, range 62–345), of which 1/9 (11.1%) were vaccinated with Moderna mRNA-1273 and 8/9 (88.8%) with Pfizer/BioNTech BNT162b2.

Blood and serum samples were obtained from all women at time of enrollment, after diagnosis of SARS-CoV-2 infection via nasal swab testing (median days since diagnosis of infection: 2; range 0–20). Cord blood samples were collected at delivery from 17 newborns from 17 mothers (n = 6 from vaccinated mothers; n = 11 from non-vaccinated mothers). The study was approved by the local Ethics Committee (P-20200046007), and all subjects gave written informed consent.

### 2.2. Quantification of Cytokines

Serum concentrations of cytokines, chemokines and growth factors were measured in duplicate using BioPlex Pro Human Cytokine Screening Panel (27-Plex #M500KCAF0Y, Bio-Rad, Hercules, CA, USA) according to the manufacturer’s instructions. Data were obtained with BIO-PLEX manager software 6.0.

### 2.3. Antibody Response

Serum samples from mothers and newborns were tested for SARS-CoV-2 Anti-Spike (S) and anti-Nucleocapsid (NCP) IgG antibodies using ELISA (Euroimmun, Lübeck, Germany), according to the manufacturer’s instructions. The semi-quantitative results were expressed as a ratio (RU/mL) with respect to an internal calibrator: a ratio of <0.8 was considered negative, ≥1.1 was considered positive and intermediate results were considered borderline.

### 2.4. PBMC Isolation

Peripheral blood mononuclear cells (PBMCs) were isolated through standard density gradient centrifugation from heparin-treated blood using Lymphoprep (Sentinel Diagnostics, Milano, Italy). PBMCs were suspended in 10% dimethyl sulfoxide (DMSO) (Corning, NY, USA) and 90% heat-inactivated fetal bovine serum (FBS, Sigma, St. Louis, MO, USA) and stored in liquid nitrogen.

### 2.5. T-Cell Response (IFNγ Production)

ELISpot assay was used to evaluate Spike-specific T-cell response (IFNγ production), according to the following protocol (Cassaniti et al., CMI 2021). Briefly, peripheral blood mononuclear cells (PBMCs) at a concentration of 2 × 10^5^/100 μL culture medium per well were stimulated for 24 h in 96-well plates (coated with anti-IFN-γ monoclonal capture antibody) with peptide pools (15 mers, overlapping by 10 amino acids, Pepscan, Lelystad The Netherlands) representative of the Spike protein (S), at a final concentration of 0.25 µg/mL. Phytoheamagglutinin (PHA; 5 µg/mL) was used as positive control and medium alone as negative control. Responses ≥ 10 net spot forming cells (SFCs)/million PBMCs were considered positive based on background results obtained with negative control (mean SFC + 2SD).

### 2.6. T-Cell Proliferative Response

To evaluate antigen-specific CD4^+^ and CD8^+^ T-cell proliferative response, PBMCs (600.000/200 µL culture medium per well) were stimulated in triplicate in 96-well round-bottom plates with SARS-CoV-2 and human actin peptide pools at a final concentration of 0.1 µg/mL. The peptide pools (15 mers, overlapping by 10 amino acids, Pepscan, Lelystad, The Netherlands) were representative of the S and NCP proteins, while a peptide pool of human actin (15 mers, overlapping by 10 amino acids, Pepscan, Lelystad, The Netherlands) was used as a negative control. After culture for 7 days in medium RPMI 1640 (Euroclone, Milano, Italy) supplemented with 2 mM L-glutamine (Euroclone), 100 U/mL penicillin and 100 µg/mL streptomycin solution (Euroclone), 10% heat-inactivated human serum AB (Sigma), 1 mM Sodium Pyruvate (Gibco, Grand Island, NY, USA), 100 mM non-essential amino acids (Euroclone) and 50 mM 2-Mercaptoethanol (Gibco) cells were washed and stained.

Briefly, cells were washed, stained with Live/Dead Fixable Violet Dye (Invitrogen) for 30 min at 4 °C and subsequently with CXCR5 BLR-1 (R&D, Minneapolis, MN, USA) for 20 min at room temperature. Cells were washed and incubated with anti-mouse IgG2b, biotinylated (Southern Biotech, Birmingham, AL, USA) and successively incubated with CD8 FITC (BD Bioscience, Franklin Lakes, NJ, USA), CD4 APC Cy7 (BD Bioscience), CD278 (ICOS) APC (Invitrogen, Waltham, MA, USA), CD25 PECy7 (BD Bioscience), CD196 (CCR6) PerCP/Cy5 (Biolegend, San Diego, CA, USA) and CD183 (CXCR3) PE (Biolegend) for 20 min at room temperature. Finally, cells were washed and resuspended in PBS 1% paraformaldehyde. Flow cytometry analyses were performed with a FACS Canto II flow cytometer and BD DIVA software (BD Biosciences). T-helper subsets (T_h_) were defined as CD25^+^ICOS^+^CXCR3^+^CD4^+^ for T_h_1, CD25^+^ICOS^+^CXCR3^-^CD4^+^ for T_h_2 and CD25^+^ICOS^+^CCR6^+^CD4^+^ for T_h_17. A cell proliferation index (CPI) for S- and NCP-specific expanded T cells was determined by subtracting the percentage of CD25^+^ICOS^+^CD4^+^ or CD25^+^ICOS^+^CD8^+^ detected in PBMCs incubated with actin peptides from the percentage of CD25^+^ICOS^+^ T-cell subsets detected in PBMCs incubated with SARS-CoV-2 peptides. A CPI > 1.5 was considered positive.

### 2.7. Statistical Analysis

Descriptive statistics for quantitative data was reported as median and range. Comparison between two groups was performed using the Mann–Whitney U-test, and correlation was calculated with Spearman’s method. All tests were two-tailed, and *p* value < 0.05 was considered statistically significant. GraphPad Prism 6.0 (GraphPad Software, La Jolla, CA, USA) was used for analyses. To draw the heatmap, we used GraphPad Prism 8.0 and Python 3.8.5 in the Jupyter notebook environment. In this case, we used “pandas” and “seaborn” modules. To calculate the factor analysis, we used Python 3.8.5. FactorAnalyzer. FactorAnalyzer was applied to statistically analyze the data in the Jupyter notebook. Factors were selected based on Eigenvalues greater than 1. Cytokines that were more than 50% correlated with each factor were classified as highly correlated. 

## 3. Results

### 3.1. Characteristics of the Pregnant Women Analyzed

As reported in Table 1, no difference in age, race, co-morbidities, clinical parameters, symptoms and therapy between vaccinated and non-vaccinated women was observed. Four women (16%) had a symptomatic infection and two of them, showing fever and cough associated with rhinitis or pharyngitis, were treated with azitromicin and steroid (betamethasone). The remaining 21/25 women (84%) had no symptoms. Of all 25 patients, 2 (8.0%) had a previous SARS-CoV-2 infection eight and sixteen months before enrollment. All the newborns (NBs) were negative for SARS-CoV-2 RNA in nasal swabs collected after birth and without neonatal complications.

### 3.2. Cytokine Production

**Figure 1:**
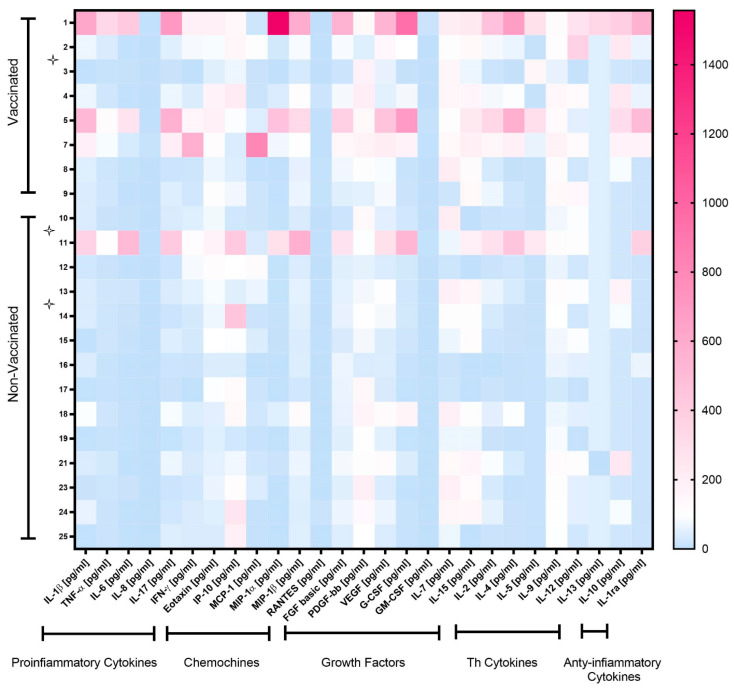
Heatmap of cytokine levels in plasma of vaccinated and non-vaccinated pregnant women with SARS-CoV-2 infection. Cytokine levels are expressed after normalization, as follows: for each cytokine, the mean concentration value was set at 100 (color white). Cytokine levels > 100 are shown in red shades, while levels < 100 are in blue shades. Symptomatic mothers are signed with star symbols.

**Figure 2:**
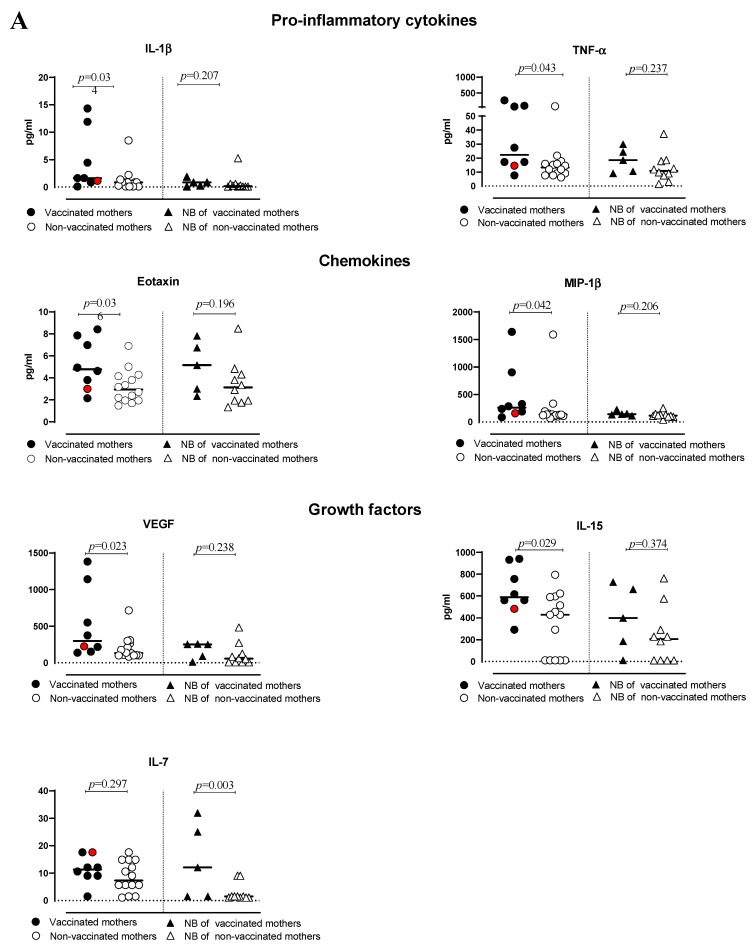

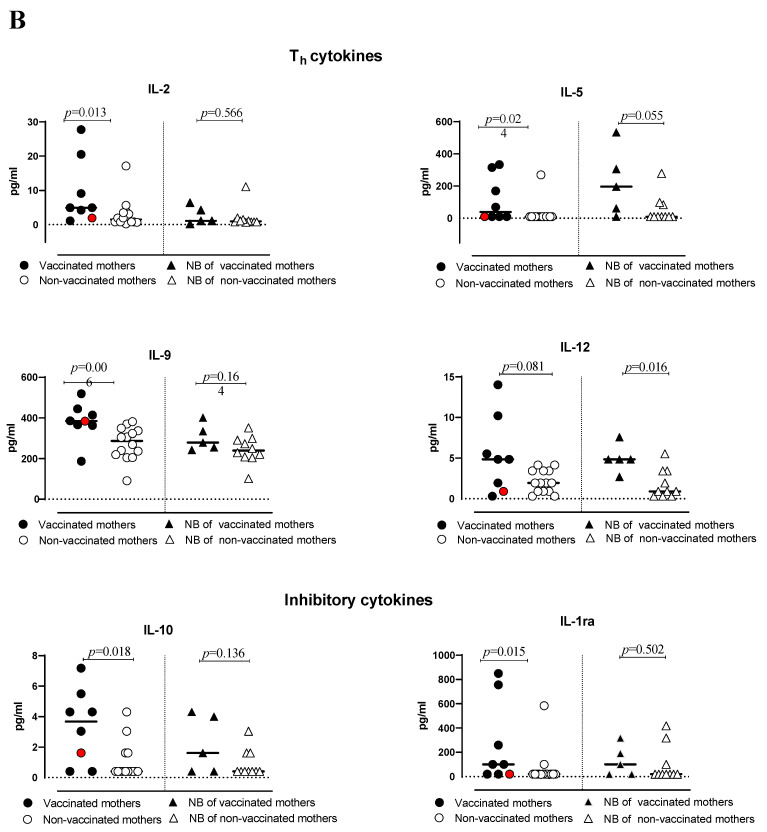
(**A**,**B**) levels of cytokines and chemokines in plasma of vaccinated or non-vaccinated pregnant women with SARS-CoV-2 infection and their newborns. Red dots indicate the women with a previous SARS-CoV-2 infection.

The serum of vaccinated/non-vaccinated mothers and their NBs was tested to evaluate the concentration and correlation of 27 different cytokines, chemokine and growth factors. In the vaccinated women, the network of cytokines was intact. Only one chemokine, RANTES (CCL5), showed a lower correlation with other cytokines, except for IP-10 (Figure S1A). In the non-vaccinated women, there was no correlation between some of the important chemokines such as IP-10 (CXCL10) with other important chemokines, cytokines and growth factors (Figure S1B). The virus even dismantles the cytokine network with disabling growth factors, such as IL-7 and PDGF-bb. The infection manipulates the cytokine network by affecting T-cell cytokines, IL-13 and correlation with other key cytokines and chemokines. In addition, the cytokine serum concentration was higher in vaccinated than non-vaccinated mothers but not symptomatically (Figure 1). In particular, IL-1β, TNF-α, Eotaxin, MIP-1β, VEGF, IL-15, IL-2, IL-5, IL-9, IL-10 and IL-1ra levels were significantly higher in vaccinated than non-vaccinated mothers. Moreover, the NBs of the vaccinated mothers produced significantly (or close to significance) higher levels of IL-7, IL-5 and IL-12 compared to the NBs of non-vaccinated mothers (Figure 2A,B). No significant difference for the other cytokines was observed. A correlation in the concentration of TNF-α, IL-6, IL-17, MIP-1α and G-CSF between non-vaccinated mothers and their NBs was noted, as reported in Table 2, but no correlation in the group of vaccinated mothers and their NBs was observed.

The non-vaccinated participants were categorized into four factors based on Eigenvalues. In the first factor, Eotaxin (CCL11, CCL24, CCL26), FGF, G-CSF, GM-CSF, IFN-γ, IL-1β, IL-1ra, IL-2, IL-4, IL-5, IL-6, IL-7, IL-8, IL-9, IL-10, IL-12, IL-15, IL-17, MCP-1/CCL2, MIP-1α/CCL4, MIP-1β/CCL3, RANTES/CCL5, TNF-α and VEGF were highly correlated. In the second factor, IL-7 and PDGF-bb/PDGFB were highly correlated, while in the third and fourth factors, MIP-1β/CCL3 and IL-13 were highly regulated, respectively, Table S1. The vaccinated pregnant women were categorized into five factors based on Eigenvalues, IL-13, eotaxin (CCL11, CCL24, CCL26), FGF, G-CSF, GM-CSF, IL-1β, IL-1ra, IL-2, IL-4, IL-5, IL-6, IL-7, IL-10, IL-15, IL-17, MIP-1α/CCL4, MIP-1β/CCL3, RANTES/CCL5, TNF-γ and VEGF were highly correlated. While in the second factor, IL-8, IL-17, MCP-1/CCL2 and IL-9 were correlated. In the third factor, IP-10 and RANTES/CCL5 were correlated. The fourth factor was highly correlated with PDGF-bb/PDGFB and IL-12. Finally, the fifth factor was correlated with IL-13, Table S2.

### 3.3. Antibody Response

**Figure 3:**
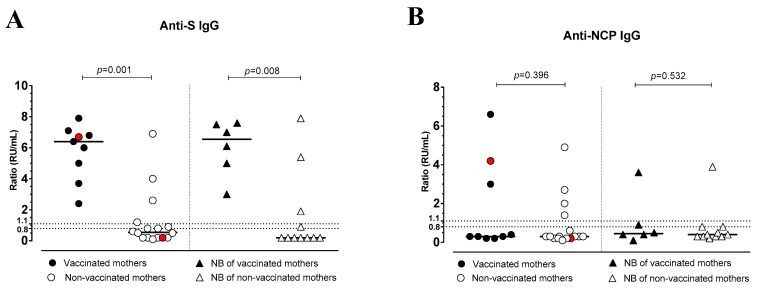
Anti-Spike (S) IgG (**A**) and anti-nucleocapsid (NCP) IgG (**B**) levels in vaccinated or non-vaccinated pregnant women with SARS-CoV-2 infection and their newborns. Red dots indicate the women with a previous SARS-CoV-2 infection.

All vaccinated mothers and their newborns showed detectable levels of anti-S IgG (median ratio: 6.4, range 2.4–7.9 in the mothers, and 6.5, range 3.0-7.6 in the NBs, respectively), which were higher compared to non-vaccinated mothers and their NBs (median ratio: 0.55, range 0.1–6.9 and: 0.2, range 0.2–7.9, respectively), among whom only 4/16 (25%) women and 3/11 (27%) NBs showed anti-S IgG. Two women had a previous SARS-CoV-2 infection before the current pregnancy, but only one (vaccinated) showed anti-S and -NCP IgG antibodies. Therefore, the anti-S IgG levels of vaccinated mothers and their NBs were significantly higher than the levels of non-vaccinated mothers (*p* = 0.0002) and their NBs (*p* = 0.0108) (Figure 3A). There was no significant difference between anti-NCP IgG levels in vaccinated mothers compared to antibody levels in non-vaccinated mothers and between the NBs of both groups (*p* = 0.396 and *p* = 0.532, respectively; Figure 3B).

### 3.4. SARS-CoV-2-Specific T-Cell Response (IFNγ Production)

**Figure 4:**
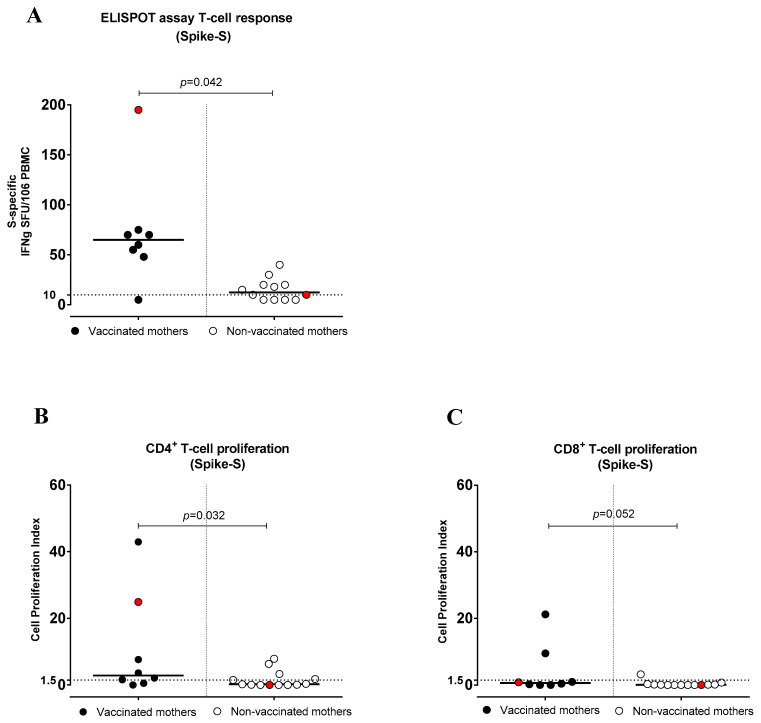
Anti-Spike (S) T-cell response in vaccinated and non-vaccinated pregnant women with SARS-CoV-2 infection. (**A**) S-specific T-cell frequency by ELISpot assay (IFNγ production), (**B**) CD4^+^ T-cell proliferation index and (**C**) CD8^+^ T-cell proliferation index. Red dots indicate the women with a previous SARS-CoV-2 infection.

T-cell frequency was quantified via ELISpot assay in 8 vaccinated and 12 non-vaccinated women. All vaccinated women (except one) showed S-specific T cells, and their frequency was significantly higher (*p* = 0.042) (median frequency: 70 SFC/million PBMCs, range 48–195) compared to non-vaccinated women (median frequency: 15 SFC/million PBMCs, range 5–40) (Figure 4 and Table S3).

### 3.5. SARS-CoV-2-Specific CD4^+^ and CD8^+^ T-Cell Proliferative Response

The vaccinated mothers tested (n = 8) showed higher levels of S-specific CD4^+^ T-cell proliferative responses (median frequency, CPI: 3.61, range 0.51–42.95; *p* = 0.032) than non-vaccinated mothers (n = 13) (median frequency, CPI: 0.27, range 0.0–7.89). Overall, 6/8 (75.0%) vaccinated women and 5/13 (38.4%) non-vaccinated women showed a CD4^+^ T-cell response. In addition, the S-specific CD8^+^ T-cell proliferative response was higher in vaccinated mothers, and the difference was close to significance (*p* = 0.052). It was detected in 2/8 (25.0%) vaccinated and 1/13 (7.7%) non-vaccinated mothers (Figure 4B,C). Among T-helper subsets, the response was restricted to CD4^+^ T_h_1 in both vaccinated and non-vaccinated women (Supplementary Figure S2A). Instead, no S-specific CD4^+^ T-cell proliferative response was observed in the T_h_2 and T_h_17 subsets (Supplementary Figure S2B,C and Table S3).

## 4. Discussion

In this study, we evaluated: (i) cytokine profile, (ii) anti-S and anti-NCP IgG antibody levels and (iii) S-specific T-cell response in a cohort of vaccinated and non-vaccinated pregnant women experiencing SARS-CoV-2 infection.

We reported different levels of pro-inflammatory or anti-inflammatory cytokines, chemokines, growth factors and T-helper (T_h_) cytokines between vaccinated and non-vaccinated mothers and their NBs. According to Figure S1, the high correlation of cytokines decreases the severity of COVID-19 in vaccinated mothers. In addition, the RANTES rise was in parallel with an increase in the secretion of IL-10 and IL-1α [24]. 

Interestingly, our results, in parallel with Buszko and colleagues [25], demonstrated that in SARS-CoV-2-infected patients, there is no correlation between IP-10 and IFN-γ. Escalation of the IP-10 is correlated with the severity of disease symptoms and the persistence of COVID-19 after infection. COVID-19 even impacts the cytokine network by accelerating the production of the cytokines, such as PDGF-bb and IL-7.

Bronchial fluids contain PDGF in COVID-19 patients [26]. The role of the PDGF is to recruit thrombocytes and mast cells to the upper part of the respiratory system [27]. In addition, IL-7 plays a critical role in refreshing the exhausted T cells in the immune responses. IL-7 affects IFN-γ production and T-cell proliferation during SARS-CoV-2 infection. IL-13 orchestrates the M2 macrophages, eosinophils, mucosal production by epithelial cells, fibrosis and metaplasia of the cells in the lower and upper part of the respiratory system [28]. In patients with severe symptoms, COVID-19 manipulates the immune cells to produce a higher amount of IL-13. The heatmap in Figure S1B demonstrates a lack of correlations and an interrupted correlation between IL-13 and the other main cytokines. However, since symptomatic women were limited in number, we could not analyze the potential associations between cytokine profile and severity of infections.

In addition, the serum concentration of the cytokines belonging to the different classes analyzed was higher in vaccinated mothers compared to non-vaccinated mothers. This phenomenon is likely to be a consequence of a prompter response to SARS-CoV-2 infection in vaccinated women. Conversely, only higher levels of IL-7, IL-5 and IL-12 were noted in the NBs of vaccinated mothers than in those of non-vaccinated mothers. The elevated levels of cord serum cytokines are probably derived directly from the newborns in response to SARS-CoV-2 maternal infection rather than from maternal transfer [9]. These may explain the different cytokine profiles observed in NBs and their mothers.

During SARS-CoV-2 infection, elevated levels of both pro-inflammatory and anti-inflammatory cytokines have been reported in multiple clinical studies [29,30,31]. In particular, the role of IL-6 along with other cytokines, including IL-10, IL-2, IL-4, IP-10, CXCL8, IL-1β, TNF-α and IFN-γ, as prognostic parameters for severe disease was observed [32,33,34,35].

In a recent study on SARS-CoV-2 infection during pregnancy, the maternal plasma cytokine analysis revealed significantly elevated IP-10 and IL-1β levels in mothers with recent or ongoing SARS-CoV-2 infection compared to mothers who recovered from previous infection, while IL-10, CXCL8 and IL-6 levels were similar between the two groups. Moreover, neonates born to mothers with recent or ongoing infection express higher plasma levels of IL-10 and CXCL8 than their paired mothers [9]. 

However, we did not observe a clear difference in the cytokine profile in NBs to vaccinated or non-vaccinated mothers with SARS-CoV-2 infection, as instead observed in maternal serum. The vaccinated mothers presented a constructed network of cytokines compared to non-vaccinated mothers, resulting from an already existing immunological response. 

The higher levels of cytokines observed in our group of vaccinated women experiencing SARS-CoV-2 infection during pregnancy are in agreement with recent studies that associated high cytokine production with improved antibody responses to SARS-CoV-2 infection occurring after the administration of the vaccine [36,37]. In particular, in addition to pro-inflammatory cytokines, we also observed a higher level of anti-inflammatory cytokines (IL-10 and IL-1ra), which may contribute to counteracting and containing the inflammatory response to the infection.

Moreover, based on the results, we analyzed each factor closely for its biological process. Utilizing the “ENRICHER” system biology (https://maayanlab.cloud/Enrichr/, accessed on 1 January 2023) helped us to analyze the biological process behind the different factors. In non-vaccinated women, the first factor was mostly taking part in eosinophil migration and chemotaxis. The second factor has a key role in glomerular mesangial cell proliferation. The cells play key roles in IL-1 and PDGF production [38]. The third-factor main biological process is positively regulating the NK cells’ chemotaxis. In addition, the fourth factor is involved in the regulation of complement-dependent cytotoxicity. In the vaccinated pregnant women, the first factor has the same function as the first factor in non-vaccinated participants, eosinophil migration and chemotaxis. The second factor has a key role in inflammatory responses. The third factor positively regulates the NK cells’ chemotaxis process. The fourth factor affects glomerular mesangial cell proliferation. The fifth factor regulates complement-dependent cytotoxicity.

Based on factor analysis in the vaccinated pregnant women, the second factor that takes part in inflammatory responses is different from non-vaccinated women. Due to angiotensin-converting enzyme 2 (ACE2) augmentation in pregnant women’s lungs, they show fewer symptoms after SARS-CoV-2 infection. Increasing the frequency of ACE2 in the lungs of pregnant women reduces the inflammatory responses in their lungs [39]. In addition, the number of plasmacytoid dendritic cells (pDCs) is decreased in pregnant women. pDCs are critically important for inflammatory responses [40]. Furthermore, the phagocytosis phenomenon of neutrophils and monocytes is decreased in pregnant women. The consequence of phagocytosis reduction is a lower production of cytokines that take part in inflammatory responses. Moreover, steroidal hormones, such as estradiol (E2) and progesterone (P4), suppress the inflammatory responses in pregnant women [41]. Thus, reduced inflammatory responses in pregnant women favor the virus in a tug of war with immune responses. As our factor analysis demonstrates, a similar reduction in inflammatory responses happens in non-vaccinated pregnant women. In contrast, in the vaccinated pregnant women, inflammatory responses are intact. This helps the vaccinated women overcome the virus far better than non-vaccinated participants. Inflammatory responses in vaccinated pregnant women are probably a result of an increased number of effector, memory and naïve CD4 and CD8 T cells. SARS-CoV-2 modifies the T cells and reduces different subtypes of T cells [42,43]. An increase in mesangial cell proliferation that is involved in producing pro-inflammatory cytokines, such as TNF, IL-1 and IL-6, is due to the study period, between 2022 January and February. Omicron BA.1 or BA.2 was circulating, causing very mild illness or asymptomatic infections, even in non-vaccinated subjects. Specifically, the breakthrough infection caused mild or asymptomatic infection in vaccinated women, but it caused a booster immunological response with trained innate immunity induced by vaccination.

Regarding the adaptive immune response, we observed higher serum anti-S IgG levels, as well as S-specific T-cell frequency and proliferative response in vaccinated than non-vaccinated mothers, as also reported in other studies [37]. In addition, we observed the predominance of a CD4^+^ T_h_1 profile in vaccinated women, in agreement with the literature. In particular, while all vaccinated women showed the presence of specific IgG antibodies, and almost all specific T cells, the majority of non-vaccinated women did not show detectable anti-S antibodies or T cells. Women were tested very close to the infection diagnosis after a median time of 2 (0–20) days; we observed, in a previous study of immune response to SARS-CoV-2 infection in the pre-vaccination era, that most infected pregnant women did not show anti-S or anti-NCP IgG within this time frame [44]. Therefore, the presence of both IgG antibodies and T cells against S in the women experiencing SARS-CoV-2 infection after vaccination could be due to the persistence of a vaccine-elicited immune response or to a more rapid response occurring in vaccinated women or both. The cellular immunity following infection or vaccination appears to remain robust and sustained [45], unlike the antibody response that tends to decrease. However, notwithstanding the decrease in antibody levels, memory B cells persists at a stable level for months after vaccination [46], therefore, being able to generate rapidly new antibody-producing cells after a re-challenge by SARS-CoV-2 infection. Of note, the single non-vaccinated women with a previous SARS-CoV-2 infection did not show anti-S IgG antibodies or T cells early after the new infectious episode, conversely to what is instead observed in the vaccinated group. This may suggest that, in some cases, the immunological memory induced by natural infection vaccination may be less sustained than that induced by the mRNA vaccine [47].

All the enrolled women had an asymptomatic or mildly symptomatic infection; therefore, our study cannot provide information about the potentially different clinical manifestation of SARS-CoV-2 infection occurring in vaccinated rather than non-vaccinated pregnant women. However, a clear advantage observed in vaccinated pregnant women developing SARS-CoV-2 infection in the last part of pregnancy is the trans-placental transfer of maternal IgG to the newborn, which was observed in all NBs from vaccinated women and in less than one-third of the newborns from non-vaccinated women. These antibodies may contribute to the protection of NBs in the case of SARS-CoV-2 infection in the first months of life.

As reported in many studies, SARS-CoV-2-specific IgG antibodies are transferred across the placenta to the neonates from their mothers, following SARS-CoV-2 infection [48,49,50]. In particular, women infected in the second trimester (13–26 weeks of gestation) developed antibodies that remained elevated at delivery [19].

Robust IgG levels were noted in all vaccinated pregnant women, and vaccine-induced IgG was transferred to the fetus, as has been noted in the setting of influenza, pertussis and other vaccinations in pregnancy [51,52]. De Rose and colleagues reported a good maternal immune response, as well as the transfer of maternal antibodies to confer passive protection against SARS-CoV-2 in newborns following maternal vaccination [53,54]. Moreover, maternal immunization during the early third trimester (27–31 weeks of gestation) yielded higher neonatal antibody concentrations, compared with the late third trimester (32–36 weeks of gestation) [55,56]. The transfer of maternal antibodies was also noted in breast milk of vaccinated mothers, suggesting a possible specific protective effect on NBs [23,37].

In our study, most non-vaccinated mothers infected in the late third trimester did not transfer IgG antibodies to the NB, likely because they could not develop a sufficient IgG antibody response early after infection, conversely to what observed in the group of infected women that were vaccinated in the preconceptional time or first trimester.

The limitations of this study are the small sample size, due to limited recruitment time, which was not sufficient to compare the clinical manifestation of SARS-CoV-2 infection in vaccinated or non-vaccinated pregnant women, and the lack of follow-up, in order to study and compare the kinetics of the immune response in the two groups. Due to the low sample size, the small p-values obtained in some analyses should be considered with caution.

In conclusion, the early development of antibodies and T-cell responses induced by previous vaccination, but also the rapid production of anti-inflammatory cytokines, could strengthen the immune and inflammatory response after infection. Furthermore, the maternal IgG antibody trans-placental transfer after SARS-CoV-2 infection is favored in previously vaccinated pregnant women and may protect NBs for several months.

## Figures and Tables

**Table 1:** Clinical characteristics of the 25 mothers.

	Vaccinated (n = 9)	No Vaccinated (n = 16)
Age at delivery, median [range]	33.0 [26.0–40.0]	32.0 [19.0–43.0]
Caucasian n. (%)	5 (55.5)	9 (56.2)
Co-morbidities n. (%)	5 (55.5)	7 (43.7)
Gestational weeks at infection, median [range]	39.0 [21.0–41.0]	38.5 [16.0–42.0]
**Haematological and biochemical parameters**		
Lymphocytes, 10^6^ cells/µL, median [range]		0.20 [0.3–7.26]
C-reactive protein, median [range]		0.09 [1.28–7.04]
AST/ALT, median [range]		26.0/19.0 [14.0–89.0/9.0–146.0]
**Symptoms:**		
Fever n. (%)	1 (11.1)	3 (18.7)
Cough n. (%)	1 (11.1)	1 (6.2)
Pharyngitis n. (%)	0 (0.0)	1 (6.2)
Rhinitis n. (%)	1 (11.1)	0 (0.0)
**Mode of delivery:**		
Vaginal n. (%)	6 (66.6)	12 (75.0)
Cesarean section n. (%)	3 (33.3)	4 (25.0)
**Therapy:**		
Azitromicin	1 (11.1)	0 (0.0)
Steroid	0 (0.0)	1 (11.1)
**Vaccine**	2 dose	\

**Table 2:** Cytokine production in vaccinated and non-vaccinated mothers and their newborns. TNF = Tumor Necrosis Factor; IFN = Interferon; IL = Interleukin; IP = Interferon gamma-inducible protein; MCP = Monocyte Chemotactic Protein; MIP = Macrophage Inflammatory Protein; RANTES = Regulated on Activation, Normal T-cell expressed, and Secreted; FGF = Fibroblast Growth Factor; PDGF = Platelet derived Growth Factor; VEGF = Vascular Endothelial Growth Factor; G-CSF = Granulocyte Colony-Stimulating Factor; GM-CSF = Granulocyte–Macrophage Colony Stimulating Factor.

	Vaccinated Mothers (Median, Range)	Non-Vaccinated Mothers (Median, Range)	*p* Value	New Borns of Vaccinated Mothers (Median, Range)	New Borns of Non-Vaccinated Mothers (Median, Range)	*p* Value	Vaccinated Mothers/New Borns (*p* Value; r Spearman)	Non-Vaccinated Mothers/New Borns (*p* Value; r Spearman)
**Pro-Inflammatory Cytokines**								
IL-1β	1.63 (0.10–14–34)	0.86 (0.10–8.5)	0.034	0.86 (0.10–1.87)	0.18 (0.10–5.27)	0.207	0.366; 0.564	0.862; −0.068
TNF-α	22.35 (7.63–266.10)	13.22 (6.16–78.03)	0.043	18.54 (9.060–29.94)	10.82 (1.38–37.25)	0.237	0.300; 0.615	0.046; −0.652
IL-6	14.94 (6.11–826.50)	9.835 (1250.00–1016.00)	0.259	6.30 (3.28–28.85)	3.75 (1.25–15.72)	0.206	0.450; 0.500	0.028; −0.699
IL-8	2287.00 (20.60–70573.00)	243.00 (65.35–5667.00)	0.876	480.40 (9.07–5972.00)	32.21 (3.60–9340.00)	0.420	0.450; 0.500	0.154; −0.487
IL17	7.34 (1.30–84.92)	3.81 (0.80–49.88)	0.119	4.38 (0.80–12.73)	2.06 (0.80–19.58)	0.493	0.350; 0.600	0.024; −0.714
IFN-γ	9.49 (0.2–86.77)	5.16 (0.20–17.35)	0.391	4.55 (2.85–9.94)	2.85 (0.20–12.11)	0.169	0.400; 0.564	0.061; −0.61
**Chemokines**								
Eotaxin	4.78 (2.15–8.41)	2.95 (1.49–6.90)	0.036	5.14 (2.34–7.82)	3.13 (1.34–8.49)	0.196		
IP-10	585.70 (260.10–1490.00)	720.90 (176.20–3037.00)	0.713	252.40 (203.40–775.00)	172.20 (46.67–3957.00)	0.206	0.783; −0.200	0.918; 0.042
MCP-1	120.40 (19.31–746.5)	107.30 (22.59–4941.00)	0.763	16.01 (6.58–24.42)	9.00 (4.97–90.12)	0.744	0.350; 0.600	0.395; −0.298
MIP-1α	32.91 (1.86–1557.00)	6.25 (0.86–280.90)	0.110	2.84 (0.86–8.49)	2.17 (0.46–104.30)	0.742	0.233; 0.700	0.004; −0.838
MIP-1β	261.80 (86.06–1637.00)	131.90 (67.64–1589.00)	0.042	143.90 (111.20–227.50)	116.30 (42.36–253.20)	0.206	0.683; 0.300	0.607; −0.187
RANTES	29,239.00 (1948.00–313,339.00)	8427.00 (4175.00–232,903.00)	0.266	19246.00 (1168.00–70380.00)	7243.00 (1007.00–30303.00)	0.206	0.233; 0.700	0.367; −0.321
**Growth Factors**								
FGF	20.59 (4.50–135.70)	14.18 (5.50–64.73)	0.056	19.10 (1.80–33.01)	5.47 (1.91–47.62)	0.526	0.133; 0.800	0.185; 0.455
PDGF	6158.00 (2095.00–9185.00)	4728.00 (1672.00–8636.00)	0.338	4458.00 (597.90–11803.00)	1865.00 (508.00–7109.00)	0.513	0.083; 0.900	0.427; −0.284
VEGF	298.80 (138.50–1384.00)	133.90 (83.52–716.10)	0.023	250.60 (11.26–257.10)	58.04 (6.00–481.90)	0.238	0.683; −0.300	0.386; −0.304
G-CSF	629.30 (43.91–6576.00)	163.30 (30.04–3713.00)	0.141	54.33 (12.90–171.90)	54.98 (12.90–2115.00)	1.000	0.350;0.600	0.037:−0.674
GM-CSF	0.20 (0.20–10.22)	0.20 (0.20–4980.00)	0.063	0.20 (0.20–1.78)	0.20 (0.20–2.92)	0.517	1.000; −0.111	1.000; −0.250
IL-7	11.33 (1.540–17.49)	7.36 (1.10–17.59)	0.297	12.07 (1.54–31.98)	1.54 (1.10–9.04)	0.038	0.400; 0.552	0.305; −0.355
IL-15	589.40 (291.3–941.6)	428.60 (11.00–794.60)	0.029	206.40 (11.00–762.30)	398.8 (11.00–728.60)	0.374	0.350; 0.600	0.367; −0.314
**T_h_ Cytokines**								
IL-2	4.94 (1.110–27.74)	1.52 (0.19–17.13)	0.013	1.11 (0.19–6.35)	0.95 (0.67–11.07)	0.566	0.067; 0.872	0.221; −0.423
IL-4	0.19 (0.10–7.35)	1.33 (0.10–11.31)	0.125	0.38 (0.10–0.89)	0.10 (0.10–3.75)	0.077	0.250; 0.657	0.600; −0.363
IL-5	39.71 (10.00–333.50)	10.00 (10.00–269.70)	0.024	196.00 (10.00–535.40)	10.00 (10.00–2.79)	0.054	0.950; 0.100	1.000; −0.214
IL-9	384.30 (186.8–518.50)	286.9 (90.74–380.70)	0.006	279.30 (242.30–400.70)	239.60 (101.10–350.90)	0.164	0.100; 0.894	0.448; −0.272
IL-12	4.85 (0.30–14.02)	1.94 (0.30–4.14)	0.081	4.85 (2.70–7.57)	0.90 (0.30–5.54)	0.016	0.125; 0.585	0.452; 0.341
IL-13	0.15 (0.15–1.09)	0.15 (0.15–6.15)	1.000	0.15 (0.15–0.33)	0.15 (0.15–0.15)	0.333	0.800; 0.344	0.435; −0.273
**Anti-Inflammatory Cytokines**								
IL-10	3.67 (0.40–7.18)	0.40 (0.40–3.67)	0.018	1.62 (0.40–4.31)	0.40 (0.40–3.04)	0.136	0.600; 0.289	0.300; 0.430
IL-1ra	100.30 (20.00–849.10)	20.00 (18.19–583.7)	0.015	100.30 (0.20–317.90)	20.00 (20.00–418.20)	0.502	0.200; 0.803	0.067; 0.872

## Data Availability

The data that support the findings of this study are available on request from the corresponding author. The data are not publicly available due to privacy or ethical restrictions.

## Supplementary Materials

The following supporting information can be downloaded at https://www.mdpi.com/article/10.3390/pathogens12050664/s1. Figure S1. Correlation between the different cytokines in vaccinated (A) and non-vaccinated women (B) was represented. Low and high correlations are indicated with blue and yellow colors respectively. Figure S2. S-specific CD4+ subsets Th1 (A), Th2 (B), and Th17 (C) T cell response. Red dots indicate the women with a previous SARS-CoV-2 infection. Table S1. A factor analysis of the unvaccinated participants. Based on the Eigenvalues, cytokines were classified into four factors. Table S2. Factor analysis of the vaccinated participants. Based on the Eigenvalues, cytokines were classified into five factors. Table S3. Total number of IFN-Y; producing cells for Spike peptide pool, negative control (medium) and PHA. Spike-specific CD4+, CD8+ T-cell and subsets in cell proliferation assay.
