# Supplementary material for: Inflammatory and Immune Responses during SARS-CoV-2 Infection in Vaccinated and Non-Vaccinated Pregnant Women and Their Newborns
Source: Pathogens. 2023 Apr 29;12(5):664. doi: 10.3390/pathogens12050664 (PMC10221808; doi:10.3390/pathogens12050664)
Supplement: Supplementary file 1 [file pathogens-12-00664-s001.zip › Figure S1.pdf]

A

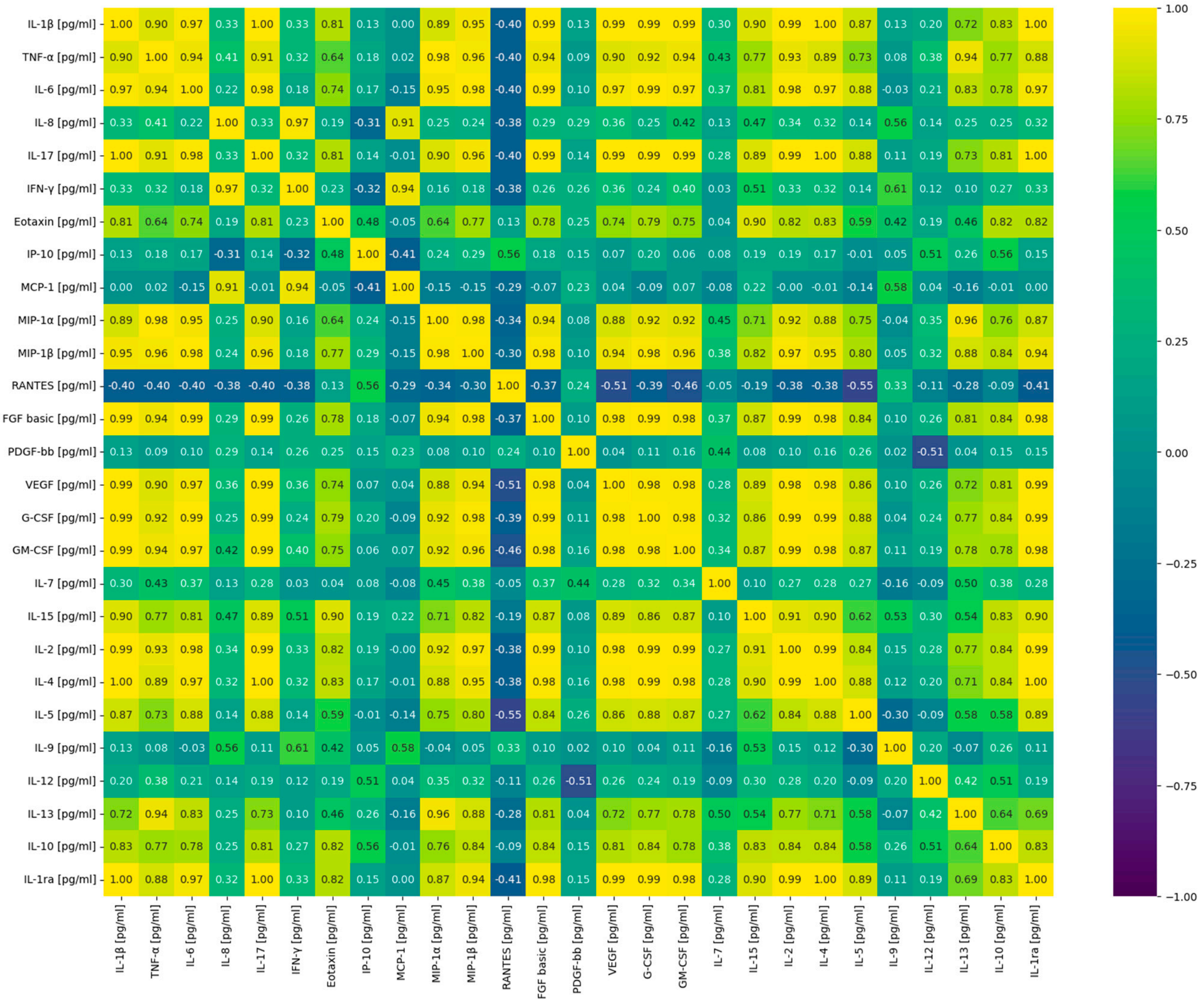

IL-1β [pg/ml]
TNF-α [pg/ml]
IL-6 [pg/ml]
IL-8 [pg/ml]
IL-17 [pg/ml]
IFN-γ [pg/ml]
Eotaxin [pg/ml]
IP-10 [pg/ml]
MCP-1 [pg/ml]
MIP-1α [pg/ml]
MIP-1β [pg/ml]
RANTES [pg/ml]
FGF basic [pg/ml]
PDGF-bb [pg/ml]
VEGF [pg/ml]
G-CSF [pg/ml]
GM-CSF [pg/ml]
IL-7 [pg/ml]
IL-15 [pg/ml]
IL-2 [pg/ml]
IL-4 [pg/ml]
IL-5 [pg/ml]
IL-9 [pg/ml]
IL-12 [pg/ml]
IL-13 [pg/ml]
IL-10 [pg/ml]
IL-1ra [pg/ml]
1.00
0.75
0.50
0.25
0.00
−0.25
−0.50
−0.75
−1.00

B

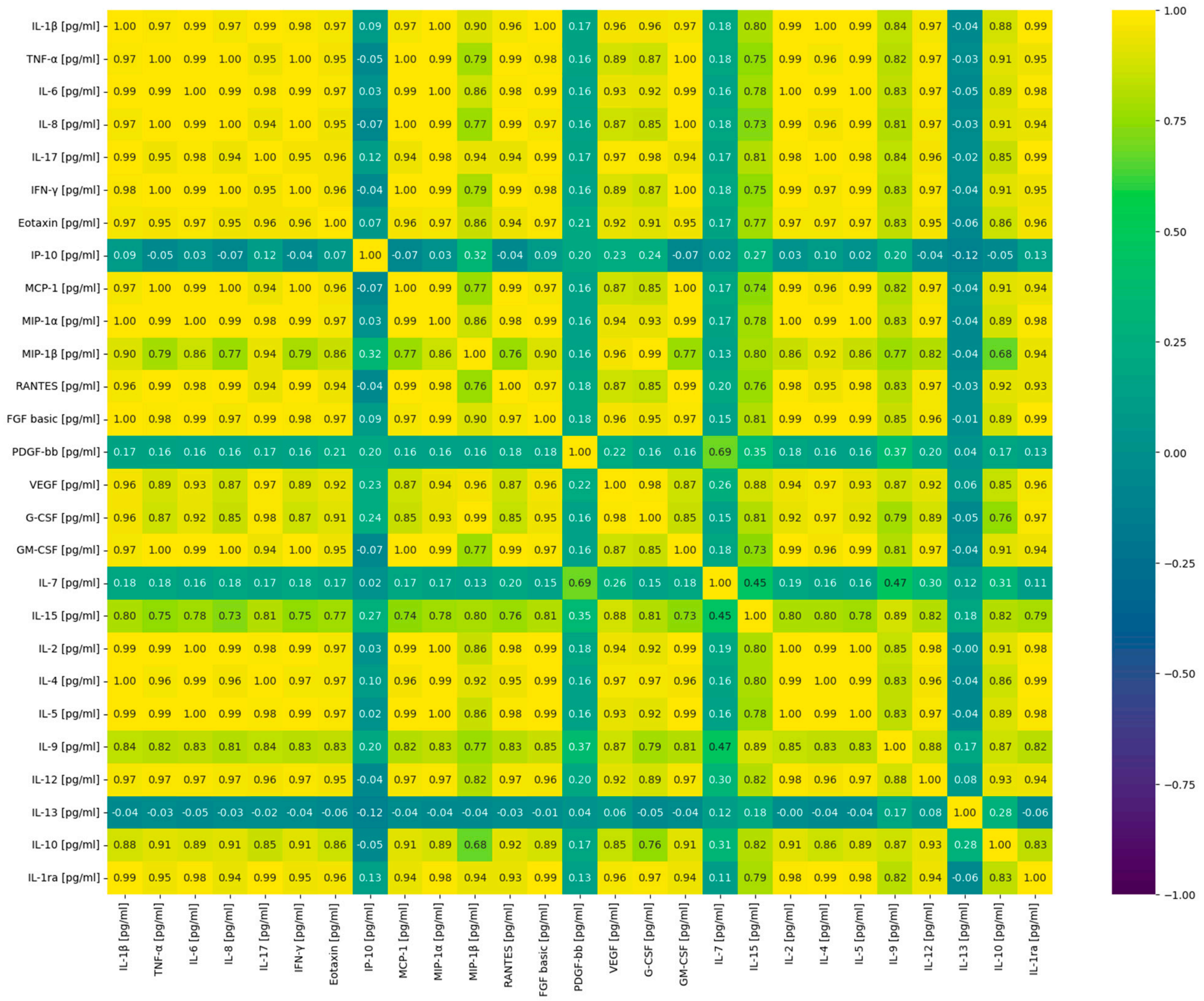

Figure S1. Correlation between the different cytokines in vaccinated (**A**) and non-vaccinated women (**B**) was represented. Low and high correlations are indicated with blue and yellow colors respectively.
