# Supplementary material for: Inflammatory and Immune Responses during SARS-CoV-2 Infection in Vaccinated and Non-Vaccinated Pregnant Women and Their Newborns
Source: Pathogens. 2023 Apr 29;12(5):664. doi: 10.3390/pathogens12050664 (PMC10221808; doi:10.3390/pathogens12050664)
Supplement: Supplementary file 1 [file pathogens-12-00664-s001.zip › Figure S2.pdf]

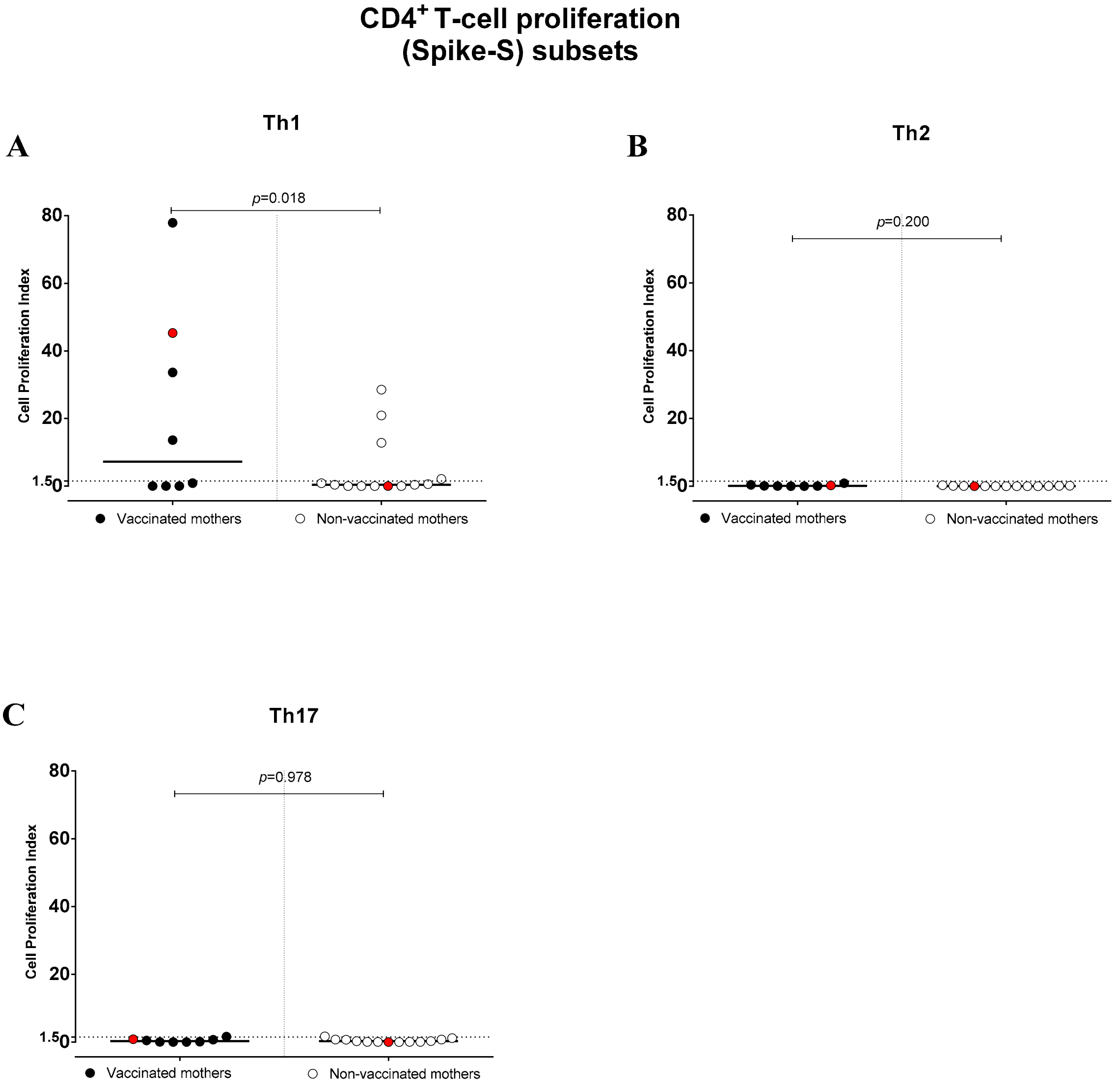

Figure S2. S-specific CD4+ subsets T_h1 (**A**), T_h2 (**B**), and T_h17 (**C**) T cell response. Red dots indicate the women with a previous SARS-CoV-2 infection.
