# Supplementary material for: Inflammatory and Immune Responses during SARS-CoV-2 Infection in Vaccinated and Non-Vaccinated Pregnant Women and Their Newborns
Source: Pathogens. 2023 Apr 29;12(5):664. doi: 10.3390/pathogens12050664 (PMC10221808; doi:10.3390/pathogens12050664)
Supplement: Supplementary file 1 [file pathogens-12-00664-s001.zip › Table S1.pdf]

Table S1. A factor analysis of the unvaccinated participants. Based on the Eigenvalues, cytokines were classified into four factors.

	1	2	3	4
IL-1β [pg/ml]	0.980065	0.087588	0.171669	-0.003461
TNF-α [pg/ml]	0.992983	0.101957	-0.049514	-0.001272
IL-6 [pg/ml]	0.993892	0.073286	0.079634	-0.010435
IL-8 [pg/ml]	0.990968	0.102075	-0.078829	-0.006593
IL-17 [pg/ml]	0.964037	0.069177	0.242886	0.027017
IFN-γ [pg/ml]	0.993407	0.105020	-0.040778	-0.004952
Eotaxin [pg/ml]	0.957408	0.112614	0.131179	-0.027690
IP-10 [pg/ml]	-0.027521	0.104799	0.653149	-0.068750
MCP-1 [pg/ml]	0.991568	0.100273	-0.078279	-0.006497
MIP-1α [pg/ml]	0.992942	0.079539	0.084002	-0.008048
MIP-1β [pg/ml]	0.819171	0.027688	0.552559	0.011517
RANTES [pg/ml]	0.981450	0.130881	-0.062608	0.011414
FGF basic [pg/ml]	0.980975	0.075718	0.165904	0.033543
PDGF-bb [pg/ml]	0.088071	0.733504	0.133005	-0.030134
VEGF [pg/ml]	0.897092	0.143839	0.384857	0.137927
G-CSF [pg/ml]	0.892582	0.048934	0.420249	-0.007527
GM-CSF [pg/ml]	0.991105	0.101818	-0.077476	-0.006725
IL-7 [pg/ml]	0.096353	0.915972	0.001591	0.123581
IL-15 [pg/ml]	0.729496	0.364363	0.358714	0.289904
IL-2 [pg/ml]	0.990289	0.102989	0.078774	0.041208
IL-4 [pg/ml]	0.975744	0.068642	0.199769	-0.002629
IL-5 [pg/ml]	0.994457	0.074762	0.070630	-0.009640
IL-9 [pg/ml]	0.790649	0.398429	0.222093	0.260062
IL-12 [pg/ml]	0.964723	0.183492	0.014919	0.139873
IL-13 [pg/ml]	-0.036499	0.057883	-0.060807	0.738208
IL-10 [pg/ml]	0.890445	0.186517	-0.074899	0.404158
IL-1ra [pg/ml]	0.965664	0.017439	0.248369	-0.014085
