# Supplementary material for: Inflammatory and Immune Responses during SARS-CoV-2 Infection in Vaccinated and Non-Vaccinated Pregnant Women and Their Newborns
Source: Pathogens. 2023 Apr 29;12(5):664. doi: 10.3390/pathogens12050664 (PMC10221808; doi:10.3390/pathogens12050664)
Supplement: Supplementary file 1 [file pathogens-12-00664-s001.zip › Table S2.pdf]

Table S1. Factor analysis of the vaccinated participants. Based on the Eigenvalues, cytokines were classified into five factors.

	1	2	3	4	5
IL-1 [pg/ml]	0.990709	0.107678	-0.020102	-0.003117	0.067078
TNF-α [pg/ml]	0.869958	0.137932	-0.032146	0.223995	0.392683
IL-6 [pg/ml]	0.974463	-0.047988	-0.054615	0.046950	0.201368
IL-8 [pg/ml]	0.219929	0.940080	-0.166644	0.010503	0.189359
IL-17 [pg/ml]	0.992638	0.095738	-0.022852	-0.011043	0.073625
IFN-γ [pg/ml]	0.226130	0.961378	-0.144989	-0.034945	0.010287
EOTAXIN [pg/ml]	0.831601	0.120035	0.511532	-0.092329	-0.187129
IP-10 [pg/ml]	0.173235	-0.268051	0.769880	0.243492	0.161133
MCP-1 [pg/ml]	-0.103566	0.970941	-0.177161	-0.044743	-0.029317
MIP-1α [pg/ml]	0.874298	-0.032380	-0.004151	0.203252	0.413111
MIP-1β [pg/ml]	0.949541	-0.014736	0.081393	0.136677	0.259806
RANTES [pg/ml]	-0.369676	-0.158544	0.850996	-0.161500	-0.046796
FGF BASIC [pg/ml]	0.978918	0.053464	0.008695	0.074556	0.166947
PDGF-BB [pg/ml]	0.085945	0.235845	0.283535	-0.753403	0.445959
VEGF [pg/ml]	0.977417	0.127180	-0.119017	0.085713	0.055265
G-CSF [pg/ml]	0.990743	0.013643	-0.004845	0.047473	0.124605
GM-CSF [pg/ml]	0.964845	0.173165	-0.093149	0.006516	0.170901
IL-7 [pg/ml]	0.247224	-0.021514	0.027557	-0.163276	0.657719
IL-15 [pg/ml]	0.884177	0.371887	0.218106	0.073525	-0.171268
IL-2 [pg/ml]	0.986033	0.113378	0.021156	0.079547	0.088137
IL-4 [pg/ml]	0.992326	0.096947	0.011100	-0.019455	0.062823
IL-5 [pg/ml]	0.881639	-0.135661	-0.281492	-0.255951	0.109929
IL-9 [pg/ml]	0.076181	0.702685	0.443662	0.099682	-0.299682
IL-12 [pg/ml]	0.190995	0.115477	0.226983	0.886034	0.066768
IL-13 [pg/ml]	0.701017	-0.035908	0.006075	0.310214	0.562932
IL-10 [pg/ml]	0.800280	0.134845	0.383631	0.203152	0.177646
IL-1RA [pg/ml]	0.991291	0.097456	-0.017272	-0.028948	0.045950
