# Supplementary material for: Inflammatory and Immune Responses during SARS-CoV-2 Infection in Vaccinated and Non-Vaccinated Pregnant Women and Their Newborns
Source: Pathogens. 2023 Apr 29;12(5):664. doi: 10.3390/pathogens12050664 (PMC10221808; doi:10.3390/pathogens12050664)
Supplement: Supplementary file 1 [file pathogens-12-00664-s001.zip › Table S3.pdf]

Table S3. Total number of IFN-Y producing cells for Spike peptide pool, negative control (medium) and PHA. Spike-specific CD4+, CD8+ T-cell and subsets in cell proliferation assay.

	Net spots/million			Cell Proliferation Index				
	S-specific IFNγ SFU/10^6	medium	PHA	$CD4^+$	T_h1	T_h2	T_h17	$CD8^+$
**Vaccinated**								
# 1	NA	NA	NA	1.61	0.00	0.42	0.04	0.23
# 2	70	0	2710	42.95	77.91	0.87	1.63	9.5
# 3	55	0	1070	7.61	33.65	0.04	0.69	0.39
# 4	75	0	1120	2.1	0.90	0.05	0.48	0.93
# 5	60	5	2050	NA	0.00	0.00	0.00	NA
# 6	48	0	2520	NA	NA	NA	NA	NA
# 7	NA	NA	NA	0.51	0,00	0.00	0.11	0.85
# 8	195	5	3000	24.94	45.29	0.23	0.86	21.2
# 9	70	5	2415	3.61	13.60	0.13	0.01	0.00
**Non-vaccinated**								
#10								
# 11	20	0	2200	0.00	0.00	0.00	0.1	0.00
# 12	5	0	2285	0.00	0.00	0.00	0.1	0.00
# 13	NA	NA	NA	NA	NA	NA	NA	NA
# 14	40	0	2620	3.3	12.83	0.08	0.7	0.1
# 15	NA	NA	NA	7.89	28.55	0.29	0.76	3.2
# 16	10	0	750	0.07	0.00	0.00	0.1	0.00
# 17	5	0	2480	0.33	2.13	0.01	0.75	0.00
# 18	15	0	1180	0.22	0.87	0.15	1.17	0.14
# 19	NA	NA	NA	1.8	0.4	0.11	0.32	0.14
# 20	NA	NA	NA	0.00	0.00	0.00	0.00	0.00
# 21	NA	NA	NA	0.00	0.00	0.00	0.00	0.00
# 22	NA	NA	NA	NA	NA	NA	NA	NA
# 23	18	0	1155	6.36	20.9	0.06	1.68	0.74
# 24	20	15	2300	1.5	0.42	0.11	0.05	0.06
# 25	5	15	2165	0.06	0.61	0.03	0.26	0.28
